# Genetic Spectrum and Characteristics of Hereditary Optic Neuropathy in Taiwan

**DOI:** 10.3390/genes12091378

**Published:** 2021-08-31

**Authors:** Chao-Wen Lin, Ching-Wen Huang, Allen Chilun Luo, Yuh-Tsyr Chou, Yu-Shu Huang, Pei-Lung Chen, Ta-Ching Chen

**Affiliations:** 1 Department of Ophthalmology, National Taiwan University Hospital, Taipei 100, Taiwan; b91401108@ntu.edu.tw (C.-W.L.); l8513437@gmail.com (C.-W.H.); amy10191911@gmail.com (Y.-S.H.); 2 Department of Medical Genetics, National Taiwan University Hospital, Taipei 100, Taiwan; allen.luo1212@gmail.com (A.C.L.); kelly.chou09@gmail.com (Y.-T.C.); 3 Graduate Institute of Clinical Medicine, College of Medicine, National Taiwan University, Taipei 100, Taiwan; 4 Graduate Institute of Medical Genomics and Proteomics, College of Medicine, National Taiwan University, Taipei 100, Taiwan

**Keywords:** hereditary optic neuropathy, next-generation sequencing, autosomal dominant optic atrophy, *OPA1*, *WFS1*

## Abstract

Hereditary optic neuropathy (HON) is a group of genetically heterogeneous diseases that cause optic nerve atrophy and lead to substantial visual impairment. HON may present with optic nerve atrophy only or in association with various systemic abnormalities. Although a genetic survey is indispensable for diagnosing HON, conventional sequencing techniques could render its diagnosis challenging. In this study, we attempted to explore the genetic background of patients with HON in Taiwan through capture-based next-generation sequencing targeting 52 HON-related genes. In total, 57 patients from 48 families were recruited, with 6 patients diagnosed as having Leber hereditary optic neuropathy through initial screening for three common variants (m.3460G>A, m.11778G>A, m.14484T>C). Disease-causing genotypes were identified in 14 (33.3%) probands, and *OPA1* variants were the most prevalent cause of autosomal HON. Exposure to medications such as ethambutol could trigger an attack of autosomal dominant optic atrophy. *WFS1* variants were identified in three probands with variable clinical features in our cohort. Hearing impairment could occur in patients with *OPA1* or *WFS1* variants. This is the first comprehensive study investigating the genetic characteristics of HON in Taiwan, especially for autosomal HON. Our results could provide useful information for clinical diagnosis and genetic counseling in this field.

## 1. Introduction

Hereditary optic neuropathy (HON) is a group of genetically heterogeneous diseases that cause optic nerve atrophy, with high variability in terms of penetrance and severity. These diseases, caused by the degradation of retinal ganglion cells, leading to a progressive, mostly irreversible deterioration of vision. Visual impairment caused by severe optic atrophy greatly hinders the patient’s everyday life. Although visual symptoms vary, most patients with HON experience visual disturbances before adolescence or in young adulthood, exerting a heavy economic burden on society.

Optic nerve atrophy could be the only manifestation of these inherited diseases. Autosomal dominant optic atrophy (ADOA) and Leber hereditary optic neuropathy (LHON) are the most common forms, with a prevalence of 1 in 30,000 to 1 in 50,000 [1,2]. However, HON may also be associated with various neurologic or systemic abnormalities. Wolfram syndrome is characterized by juvenile diabetes mellitus (DM), diabetes insipidus, sensorineural deafness, and optic atrophy [3]. GAPO syndrome combines growth retardation, alopecia, pseudoanodontia, and optic atrophy [4]. Other genetic disorders associated with optic atrophy include Warburg Micro Syndrome [5]; cerebellar ataxia, areflexia, pes cavus, optic atrophy, and sensorineural hearing loss (CAPOS) syndrome [6]; Mohr–Tranebjaerg syndrome [7]; spinocerebellar ataxia; hereditary spastic paraplegia [8]; MEGDEL syndrome [9], and Charcot–Marie–Tooth disease [10]. 

The definite diagnosis of HON is difficult, and genetic diagnosis is usually necessary. These disorders are due to genetic defects in mitochondrial genes or nuclear genes, which affect mitochondrial function [11]. This causes dysfunction of oxidative phosphorylation of the mitochondrion and subsequently damages the retinal ganglion cells, which have high metabolic demands. LHON is a mitochondrial DNA mutation–related optic neuropathy characterized by acute or subacute visual loss. In over 90% of cases, LHON is caused by three disease-causing variants (m.3460G>A, m.11778G>A, and m.14484T>C) and could be detected by Sanger sequencing [12]. However, more than 30 genes located in the nuclear chromosomes have been identified to influence mitochondria function and lead to optic atrophy [13]. Conventional genetic tests for inherited disorders of the optic nerve diseases have a limited target screening capacity and an unsatisfactory detection rate. In recent years, next-generation sequencing (NGS) has been demonstrated to be a cost-effective and time-saving technique that has revolutionized medical genetics. Some large-scale NGS-based genetic screening studies for HON have been published [14,15]. 

This study attempted to explore the efficacy and efficiency of a panel-based NGS test for diagnosing patients with suspected HON. We aimed to investigate the clinical characteristics and genetic background of patients with HON in Taiwan, especially those whose HON is unlike typical LHON. To the best of our knowledge, no similar investigation has been conducted in Taiwan.

## 2. Materials and Methods

### 2.1. Participant Enrolment

This study conducted at National Taiwan University Hospital adhered to the tenets of the Declaration of Helsinki and was approved by the Research Ethics Committee of National Taiwan University Hospital (NTUH-REC No. 201508063RINB). Informed consent was obtained from all the participants prior to enrolment. From March 2016 to February 2021, 57 patients from 48 families with suspected HON were recruited. The first family member patient who participated in our study was defined as the “proband.” The inclusion criteria of HON were: (i) the presence of optic atrophy on fundus examination or obviously reduced thickness of retinal nerve fiber layer, as analyzed using optical coherence tomography (OCT); (ii) decreased visual acuity or visual field defect (or both); (iii) onset at childhood, adolescence, or young adulthood with chronic or slowly progressive course; (iv) exclusion of other causes of optic neuropathy such as birth insult, malnutrition, toxin-induced, infiltrative process, ischemic episode, optic neuritis, glaucoma, compressive lesion, and trauma.

In the present project, we aimed to explore autosomal HON using a panel-based NGS platform. Patients suspected as having HON were initially screened for three common LHON disease-causing variants (m.3460G>A, m.11778G>A, and m.14484T>C). Undiagnosed patients were then recruited into our present cohort. A comprehensive history, including the disease course and pedigree was obtained for all recruited patients. Detailed ophthalmological examinations, including best-corrected visual acuity, indirect ophthalmoscopy, OCT imaging of the optic disc and retina, fundus photography, and automated visual field, were performed.

### 2.2. Capture-based Next-Generation Sequencing and Variant Filtering

The genomic DNA of the participants was obtained from peripheral blood and extracted using a DNA extraction kit (Gentra Puregene Blood Kit; QIAGEN, Hilden, Mettmann, Germany). We set up a panel containing 52 HON-related genes (Supplementary Table S1), which were selected from the OMIM database (https://www.ncbi.nlm.nih.gov/omim, access date 10 February 2016) and peer-reviewed publications (search queries PubMed: hereditary optic neuropathy, optic atrophy, gene). All exons (including the 5′ and 3′ untranslated regions) with at least 100-bp flanking intron sequences were defined as targeted regions. We also captured the entire genomic sequence, including both exons and introns for some genes (*NR2F1*, *OPA1*, *OPA3*, *MFN2*, *SLC25A4*, *C10orf2*, *POLG2*, *TYMP*, *C12orf65*, *TMEM126A*, *PLA2G6*, *C19orf12*, *POLG*, *SETX*, *SLC52A2*, *CYP7B1*, *SLC25A46*, *HEXB*, *PARL*, *SCN2A*, *TK2*, *DGUOK*, *MPV17*, and *RRM2B*) to increase the detection ability of possible intronic disease-causing variants.

Capture-based target enrichment was performed using a SeqCap EZ hybridization and wash kit (Roche NimbleGen, Madison, WI, USA). Paired-end sequencing was performed using an Illumina MiSeq or NextSeq 550 system (Illumina, San Diego, CA, USA). The capture probes were designed using NimbleDesign (https://design.nimblegen.com/, access date 1 July 2017) and produced by Roche NimbleGen. Genome build GRCh37 (Feb. 2009 GRCh37/hg19) was used for mapping and variant calling. The mapped reads reached a mean 138.78-fold coverage depth, and 96.82% of targeted regions were covered by 20 or more reads. 

Variant calling for single-nucleotide variants and small insertions/deletions was conducted using GATK software (version 3.4). ANNOVAR 2016Feb01 was used to annotate the allele frequency of variants based on the ClinVar database (https://www.ncbi.nlm.nih.gov/clinvar/, access date 1 March 2021), Genome Aggregation Database (gnomAD, https://gnomad.broadinstitute.org, access date 1 March 2021), NHLBIESP 6500 exome project (http://evs.gs.washington.edu/EVS/, access date 1 March 2021), 1000 Genomes project (http://www.1000genomes.org/, access date 1 March 2021), Exome Aggregation Consortium projects (ExAC, http://exac.broadinstitute.org/, access date 1 March 2021), 69 Genomes Data (CG69, http://www.completegenomics.com/public-data/69-genomes/, access date 1 March 2021), Kaviar Genomic Variant Database (http://db.systemsbiology.net/kaviar/, access date 1 March 2021), dbSNP Build 147 (avsnp147, https://www.ncbi.nlm.nih.gov/snp, access date 1 March 2021), and Taiwan Biobank (https://taiwanview.twbiobank.org.tw/index, access date 1 March 2021), a biobank that contains the data of 1517 community-based healthy individuals. Pathogenicity was predicted using SIFT, PolyPhen-2 HDIV, PolyPhen-2 HVAR, LRT, MutationTaster, FATHMM, Mutation Assessor, PROVEAN, VEST, MetaSVM, MetaLR, MCAP, CADD, and DANN. The TAIGenomics platform (https://taigenomics.tw/, access date 1 March 2021) and proprietary scripts were used to conduct and stream different steps of the bioinformatics pipeline. The integrative genomics viewer was used to visualize the sequence mapping. 

Variant annotation and filtering were performed per the method of a previous study [16]. In brief, synonymous variants and variants with allele frequencies higher than 5% in the abovementioned population databases were filtered out. The pathogenicity of suspected disease-causing variants was classified according to the American College of Medical Genetics and Genomics (ACMG) guidelines [17]. Every variant identified in our project was searched using the literature search engines Mastermind (https://www.genomenon.com/mastermind/, access date 10 March 2021) and variant2literature (https://variant2literature.taigenomics.com, access date 10 March 2021). Sanger sequencing was then used to verify the nucleotide change.

### 2.3. Statistical Analysis

The results were expressed as the mean ± standard deviation or median with interquartile range. Student’s *t* test was performed to compare the data. The significance threshold was *p* < 0.05. All analyses were performed using SPSS version 12.0 (SPSS, Chicago, IL, USA).

## 3. Results

### 3.1. Demographic Data of Study Participants

In total, 57 patients from 48 families suspected of having HON were recruited. The average patient age was 26 (range 3–72, median 20) years, and 35 (61.4%) patients were male. 

Among all patients except for one 65-year-old woman who had ADOA induced by ethambutol use, the average age of symptom onset was 10.6 (range 1–37, median 8) years. Among all patients, 11 (22.9%) probands had a positive family history of optic neuropathy. The average age of onset of HON was 12.8 years in patients with positive family history and 9.3 years in patients without a family history. The age of disease onset did not significantly differ between patients with a positive versus negative family history.

### 3.2. Genetic Characteristics of HON in Taiwan

**Figure 1:**
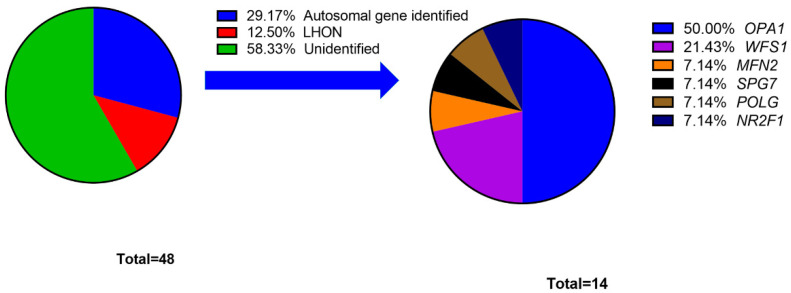
Percentage of probands grouped by hereditary optic neuropathy disease-causing gene.

A total of six patients were diagnosed as having LHON. Four patients had the variant m.11778G>A, and two patients had the variant m.14484T>C. The other 51 patients (number of probands: 42) were recruited into the cohort of individuals with autosomal HON. Through NGS-based genetic screening, disease-causing variants were identified in 14 probands, with a detection rate of 33.3%. The probands in this cohort are detailed in Supplementary Table S2. The overall diagnostic rate (autosomal HON and LHON) was 41.7%. Figure 1 lists all the disease-causing genes affecting the probands and the percentage of the probands affected in our study.

In the cohort of individuals with autosomal HON, *OPA1* was identified in 14 patients and seven probands and was the most common disease-causing gene (50%). Except for *WFS1*, other genes exhibited a pattern of autosomal dominant (AD) inheritance. Among 14 probands with disease-causing genes, we identified 17 different variants, including eight missenses, four nonsenses, and five frameshifts variants. The data of disease-causing variants are shown in Table 1, and the detailed information, including clinical manifestations, allele frequency and criteria for ACMG classification, is disclosed in Supplementary Table S2.

### 3.3. Novel Disease-Causing Variants Identified in HON Cohort

**Figure 2:**
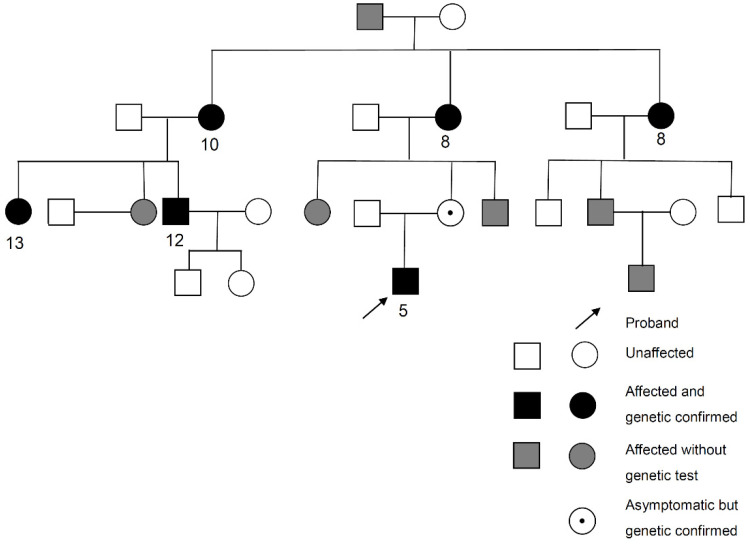
Pedigree and age of onset in each patient of Proband No. 12 family. Arrow: Proband No. 12.

**Figure 3:**
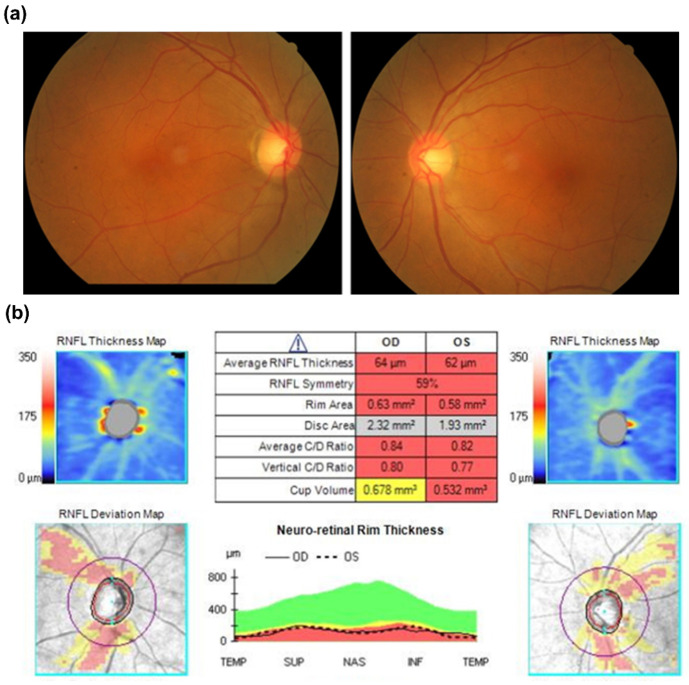
(**a**) Fundoscopic exam initially showed pinkish optic disc at disease onset. (**b**) Optical coherence tomography revealed a remarkably decreased retinal nerve fiber layer of both eyes six months after disease onset.

In our cohort, five novel variants were identified from five probands. Three of them were *OPA1* variants. The first variant (Proband No. 12) was a frameshift *OPA1* variant (p.Asp1005Ter) that affected six patients and one asymptomatic carrier, the largest family in our cohort. The pedigree and age of onset in each patient are summarized in Figure 2. The pedigrees of other cases are shown in Supplementary Figure S1. The second variant (Proband No. 9) was also a frameshift *OPA1* variant (p.Leu526ArgfsTer17), causing ADOA in a female patient, with 13 years as the age of onset. The third (Proband No. 23) was a nonsense *OPA1* variant (p.Tyr113Ter) in a 65-year-old woman. The patient was initially asymptomatic. However, she experienced progressive visual loss within 1 month after ethambutol use for extrapulmonary tuberculosis. OCT revealed severe retinal nerve fiber layer thinning in both eyes several months later (Figure 3).

The other novel variant (Proband No. 1) was a missense *MFN2* variant (p.Val69Ile). This patient has had a severe visual impairment since the age of 3 years. A fundus examination indicated salt-and-pepper retinopathy, retinal vessel sheathing, and optic atrophy in both eyes. She also exhibited developmental delay and epilepsy. Although this variant was classified as uncertain significance according to the ACMG guidelines, it had three moderate evidence of pathogenicity (PM1, PM2, PM5). Alternative variant (p.Val69Phe) was classified pathogenic and associated with Charcot–Marie–Tooth disease [18]. 

The last novel variant identified (Proband No. 22) was a pathogenic nonsense *PARL* variant (p.Arg4Ter) inherited from the patient’s mother, who had no symptoms or signs of HON. This patient also had a de novo pathogenic *OPA1* variant (p.Cys490Arg). *OPA1* and *PARL* may have some interaction in the regulation of mitochondrial morphology and apoptosis [19]. This patient had a visual impairment since the age of 3 years and progressive sensorineural hearing impairment since the age of 5 years.

### 3.4. Important Disease-Causing Genes in HON Cohort

*OPA1* was the most common disease-causing gene in our cohort. Among seven variants, two and two were identified in exons 2 and 14, respectively; each of the remaining three was located in exons 16, 28, or 30. The GTPase domain (exon 10–17) was the most frequent location of disease-causing variants. 

*WFS1* was the second most common disease-causing gene in our cohort that affected three patients. All the disease-causing variants were located in exon 8. The presentations of these three patients differed. A single disease-causing variant (p.Ala684Val) was identified in one patient (Proband No. 2). She had hearing loss since 6 months of age and progressive visual impairment at 3 years old. This variant has been reported to be a frequent cause of optic atrophy and hearing impairment [20]. The second patient (Proband No. 30) had a typical course of Wolfram syndrome, including diabetes, optic atrophy, and hearing impairment. He had impaired vision since 3 years of age. The third patient (Proband No. 3) had two *WFS1* disease-causing variants but had only optic atrophy without diabetes or hearing loss. The onset of visual loss in this patient was at the age of 34 years.

*NR2F1*, *POLG*, and *SPG7* were identified to be the disease-causing genes in a single proband. The patient (Proband No. 27) with *NR2F1* nonsense variant had visual loss and nystagmus since the age of 8 years. The patient (Proband No. 25) with *POLG* missense variant had optic atrophy since the age of 8 years, along with epilepsy. The patient (Proband No. 31) with *SPG7* frameshift variant had a visual impairment since the age of 6 years but had no symptoms of spastic paraplegia.

## 4. Discussion

The diagnosis of HON is peculiarly difficult compared to that of other inherited ocular diseases such as inherited retinal degenerations (IRDs) because of the relatively late clinical manifestation of HON and the difficulty in differentiating it from toxic, nutritional, ischemic, traumatic, and glaucomatous optic neuropathy. In addition to detailed history-taking, genetic tests play a crucial role in the diagnosis and prognostic evaluation of HON. 

HON could be inherited in AD, autosomal recessive (AR), and mitochondrial forms. LHON, a HON caused by mitochondrial DNA mutation, has its characteristic course and disease-causing hotspots. Traditional Sanger sequencing could be utilized to identify over 90% of LHON cases [12]. Therefore, in this study, we focused on exploring autosomal HON with a panel-based NGS test containing 52 HON-associated genes. Recently, two large-scale NGS-based genetic surveys for HON have been published [14,15]. Although the numbers and ranges of the target genes differed, these studies have reported a detection rate of 22–40% in identifying the disease-causing variants. Our project identified 14 disease-causing variants among 42 probands, with a diagnostic rate of 33.3%, which is comparable with those of previous studies. Previous studies have shown that AD was much more common than AR in patients with HON [14,15]. Our results also verified this observation because only two patients were classified as having AR-pattern among 14 patients with a diagnosis.

In our cohort, *OPA1* was responsible for seven probands (50% of probands with a genetic diagnosis of autosomal HON) and was the most common disease-causing gene. This percentage was similar to that observed in previous studies [15,21]. *OPA1* contains 30 exons spread over 100 Kb of genomic DNA [22]. Two missenses, three nonsenses, and two frameshifts variants were detected in our study. Haploinsufficiency is a crucial mechanism of ADOA. The GTPase domain was the most frequent location of disease-causing variants, a finding that is also compatible with those of previous studies [15,23]. Among all patients except for one patient with ethambutol-induced optic neuropathy, the mean age of onset was 8.8 years, and all patients had visual symptoms before the age of 20 years. However, the phenotypes and clinical manifestations of *OPA1* variants were highly heterogeneous, even within a single family. With the same *OPA1* disease-causing variant, one patient had severe optic atrophy with best-corrected visual acuity of 20/2000, but another patient was asymptomatic. This scarce genotype-phenotype correlation has been demonstrated in earlier studies [21,24,25]. 

Incomplete penetrance is a key characteristic in *OPA1*-related ADOA. In some circumstances, individuals with an *OPA1* variant may not develop a clinically relevant optic atrophy [26]. Environmental influence, toxin exposure, and interaction of chromosomal and mitochondria genetic backgrounds may also play some roles. In our cohort, one patient (Proband No. 23) had subacute onset visual loss 1 month after ethambutol use, which was much shorter than the usual natural course of ethambutol-related toxic optic neuropathy. Her retinal nerve fiber thickness was initially normal, but severe optic atrophy with poor visual acuity (20/400) developed within 2 months. Therefore, we performed an NGS-based genetic test for this patient, which detected a pathogenic disease-causing variant of *OPA1*. This scenario is rare but not undocumented [27]. Our finding verified that ethambutol could be a trigger for ADOA attacks.

Up to 20% of patients with *OPA1* disease-causing variants develop other systemic symptoms, such as sensorineural hearing loss, progressive external ophthalmoplegia, ataxia, or peripheral neuropathy, which usually follow ocular symptoms are collectively called the ADOA plus syndrome [28,29,30]. Among the systemic complications, bilateral hearing loss is the most common extraocular symptom [28]. Hotspots such as p.Arg445His mutations were found to be associated with sensorineural deafness and optic atrophy [21,31,32,33]. However, other variants have also been proposed in previous studies [31,32,33,34]. Some studies have hypothesized that the dominant negative effect caused by missense mutations results in the ADOA plus syndrome [30,31]. Patients with ADOA plus syndrome were more likely to have disease-causing variants in exons 14, 15, and 17 [35]. One patient (Proband No. 22) in our cohort also had ADOA plus syndrome. His visual symptoms developed when he was approximately 3 years old, and hearing loss occurred when he was 5 years old. Missense *OPA1* variant (p.Cys490Arg) in exon 14 was identified in this patient. This variant has been reported in a previous study, and hearing loss was noted in one out of two patients with this variant [34]. In addition to this variant, our patient had *PARL* nonsense variant, which was determined as pathogenic by ACMG classification. The interactions between *OPA1* and *PARL* have been proposed in a previous review article [19]. The regulatory networks comprising *OPA1* and *PARL* are implicated in the metabolism and apoptosis of mitochondria. The influence of combined *OPA1* and *PARL* mutations on the development of hearing defects in patients with ADOA warrants further investigation. 

*WFS1* was another important disease-causing gene present in a higher frequency in our cohort and was detected in three probands. Wolfram syndrome is a rare disease, with a prevalence ranging from 1 in 50,000 to 1 in 770,000 [36]. The typical manifestations of Wolfram syndrome include diabetes insipidus, DM, optic atrophy, and deafness, also termed as DIDMOAD. However, only 28% of patients express the entire DIDMOAD phenotype [37]. The inherited pattern of *WFS1* mutations could be AD or AR. The symptom onset and clinical features are also highly variable [38]. For example, the age at diagnosis of DM could range from infancy to 51 years [39]. Two of our patients had two disease-causing variants that were associated with AR disease. The first patient (Proband No. 30) had typical features of Wolfram syndrome, except for diabetes insipidus. He had developed optic atrophy, DM, and deafness before the age of 3 years. The other patient (Proband No. 3) had a relatively late onset of visual loss (34 years of age), and no DM or hearing loss was diagnosed till the time of writing this study. The second variant of this patient was classified as uncertain significance according to the ACMG guidelines but was predicted to be pathogenic in a previous publication [40]. The patient (Proband No. 2) with a single disease-causing variant (p.Ala684Val) developed hearing problems and progressive optic atrophy before the age of 3 years. This variant has been identified as a hotspot of *WFS1*-related disorder with combined optic atrophy and deafness [20]. Another finding in our cohort was that all the variants were situated in exon 8. The phenomenon of disease-causing variants concentrated in exon 8 has been observed in previous studies [36,41].

One patient (Proband No. 1) in our cohort presented with severe visual loss at the age of 3 years. Fundoscopy measurements indicated optic atrophy and salt-and-pepper retinopathy. Missense *MFN2* variant was detected and considered as the disease-causing gene. Like the *OPA1* gene, *MFN2* encodes dynamin-like GTPase proteins and facilitates the fusion of mitochondrial membrane [42]. *MFN2* could be associated with Charcot–Marie–Tooth disease type 2A and optic atrophy [42]. *NR2F1* was another gene that could be associated with optic atrophy. One of our patients (Proband No. 27) presented with visual impairment and nystagmus since he was 8 years old. He also had autism and developmental delay. Nonsense *NR2F1* variant was identified and defined as pathogenic by ACMG classification. The disruption of *NR2F1* may influence the neurodevelopment of the visual system and cause optic atrophy with intellectual disability [43].

*POLG* is reported to be associated with progressive external ophthalmoplegia, epilepsy, and optic atrophy [44,45]. *POLG* variants could express a broad phenotypic spectrum and could be inherited with an AD or AR pattern [46]. Our patient (Proband No. 25) presented with optic atrophy and epilepsy since the age of 8 years but had no symptoms of ophthalmoplegia till the age of 10 years. A Missense *POLG* variant located in exon 18 was detected. A previous study had reported that point mutations in exon 18 could result in decreased enzyme catalytic activity and DNA-binding affinity, consequently leading to disease [47]. *SPG7* is the disease-causing gene of hereditary spastic paraplegia but could occasionally lead to isolated optic atrophy [48]. One of our patients (Proband No. 31) presented with visual impairment at the age of 6 years, and he had a pathogenic *SPG7* frameshift variant. Although this variant has been reported in AR case [48], the pattern of *SPG7* inheritance is complex, including recessive, dominant, and digenic forms of inheritance [49]. Further investigation for the second variant in *SPG7*-interacting genes is feasible. Due to the broad phenotypic spectrum of disease-causing genes, some associated genes with systemic abnormalities should also be considered in isolated optic neuropathy.

Our study has some limitations. First, the number of cases in our cohort is relatively small compared with that in other large-scale NGS-based genetic surveys. However, HON is a rare disease with a much lower prevalence than IRD, and we would like to emphasize that this platform is for patients with non-LHON, as typical LHON could typically get genetically diagnosed through screening of three common variants. Second, this project was performed with a panel-based NGS genetic test that targeted exons and, partially, introns of 52 genes associated with HON. Some novel or uncommon genes may not be identifiable. Some variants with large-fragment insertion, deletion, inversion, or complicated rearrangement could also miss detection through our methods. Copy number variation was also not evaluated in our study. Third, information about symptoms, disease onset and family history, or pedigrees mainly relies on the patients’ memory, and its accuracy could be compromised. Recall bias or difficulty in approaching distant relatives could be a concern. Despite these limitations, we believe that our results still provide valuable information about the genetic characteristics of HON in Taiwan. The information could be useful for clinical diagnosis, genetic counseling, and possible gene therapy in the future.

## 5. Conclusions

This study presents a panel-based NGS genetic test platform for autosomal HON, a rare disease with diagnostic challenges. For a 5-year period, we recruited 57 patients from 48 families, and the detection rate of autosomal HON was approximately 33%. To the best of our knowledge, this is the first comprehensive study to investigate the epidemiology and genetic background of autosomal HON in Taiwan, wherein six disease-causing genes were identified. Compatible with other large-scale genetic screening studies, this study observed that *OPA1* was the most prevalent disease-causing gene, accounting for approximately 50% of probands. However, the phenotypes of *OPA1* variants exhibited high variability, and the penetrance was incomplete. Moreover, exposure to medications such as ethambutol could induce the acute attack of ADOA. Because tuberculosis still has a relatively high incidence in Taiwan, this information warrants more attention from clinical practitioners. It is noteworthy that *WFS1* variants were identified in three probands in our cohort. Wolfram syndrome could be underdiagnosed because its clinical features may vary. Hopefully, our results can elucidate HON’s genetic background and characteristics and provide comparative regional data in this field.

## Figures and Tables

**Table 1:** The list of disease-causing variants in 14 probands in our cohort.

Proband	Onset Age	Affected Gene	Coding Impact	Distribution	Variant 1	ACMG Classification	Variant 2	ACMG Classification
P01	3	*MFN2*	Missense	Exon 3	**c.205G>A (p.Val69Ile)**	Uncertain Significance		
P02	3	*WFS1*	Missense	Exon 8	c.2051C>T (p.Ala684Val)	Pathogenic		
P03	34	*WFS1*	Missense	Exon 8	c.2020G>A (p.Gly674Arg)	Likely Pathogenic	c.2194C>T (p.Arg732Cys)	Uncertain Significance
P09	13	*OPA1*	Frameshift	Exon 16	**c.1576_1577insG (p.Leu526ArgfsTer17)**	Pathogenic		
P12	5	*OPA1*	Frameshift	Exon 30	**c.3011_3012insG (p.Asp1005Ter)**	Pathogenic		
P20	3	*OPA1*	Frameshift	Exon 28	c.2873_2876delTTAG (p.Val958GlyfsTer3)	Pathogenic		
P22	3	*OPA1; PARL*	Missense; Nonsense	Exon 14; Exon 1	c.1468T>C (p.Cys490Arg)	Pathogenic	**c.10C>T (p.Arg4Ter)**	Pathogenic
P23	65	*OPA1*	Nonsense	Exon 2	**c.339C>A (p.Tyr113Ter)**	Pathogenic		
P25	8	*POLG*	Missense	Exon 18	c.2830G>A (p.Glu944Lys)	Uncertain Significance		
P26	6	*OPA1*	Missense	Exon 14	c.1499G>A (p.Arg500His)	Pathogenic		
P27	8	*NR2F1*	Nonsense	Exon 3	c.1117C>T (p.Arg373Ter)	Pathogenic		
P30	3	*WFS1*	Frameshift;Missense	Exon 8	c.1611_1624del (p.Cys537Ter)	Pathogenic	c.2336T>G (p.Val779Gly)	Uncertain Significance
P31	6	*SPG7*	Frameshift	Exon 8	c.1045_1046insC (p.Gly349AlafsTer47)	Pathogenic		
P36	8	*OPA1*	Nonsense	Exon 2	c.112C>T (p.Arg38Ter)	Pathogenic		

The variants highlighted in bold are novel variants.

## Data Availability

All data generated or analyzed during this study are included in this published article and the Supplementary Materials.

## Supplementary Materials

The following are available online at https://www.mdpi.com/article/10.3390/genes12091378/s1, Table S1: 65 genes associated with autosomal hereditary optic neuropathy. Table S2: The information of clinical manifestations, disease-causing variants identified, allele frequency, and criteria for ACMG classification for each proband in our cohort. Figure S1 Pedigree of all the probands with disease-causing gene identified.
